# The Relationship of Antibodies to Modified Citrullinated Vimentin and Markers of Bone and Cartilage Destruction in Rheumatoid Arthritis

**DOI:** 10.1155/2014/464585

**Published:** 2014-04-15

**Authors:** A. S. Avdeeva, E. N. Aleksandrova, A. A. Novikov, A. V. Smirnov, M. V. Cherkasova, E. L. Nasonov

**Affiliations:** Nasonova Research Institute of Rheumatology, Kashirskoe shosse 34 A, Moscow 115522, Russia

## Abstract

*Objective.* To make individualised decisions regarding treatment is one of the most important challenges in clinical practise, and identification of sensitive and specific markers of prognosis is an important research question. The main objective of this study was to evaluate relationships between the level of autoantibodies, radiographic changes and laboratory markers of bone, and cartilage destruction. *Methods.* A total of 114 RA patients were examined. The serum concentration of IgM RF, antibodies to cyclic citrullinated peptide (anti-CCP), modified citrullinated vimentin (anti-MCV), matrix metalloproteinase 3 (MMP-3), and cartilage oligomeric matrix protein (COMP, ng/mL) were measured. The van der Heijde-modified Sharp Score was used to quantify the radiologic changes. *Results*. Among the patients who were high-positive for anti-MCV, the value of total modified Sharp score (mTSS) (96.5; 66–120) was higher as well as the joint space narrowing (82; 60.5–105.5), and a higher level of MMP-3 was recorded more frequently (56%) in comparison with negative/low-positive patients (57; 31–88, 50; 29–82, 31% resp., *P* < 0.05). The level of COMP was also higher among patients high-positive for anti-MCV (9.7; 8.1–13.1 and 6.8; 5.4–10.7, resp., *P* = 0.02). *Conclusion.* A high positive level of anti-MCV as contrasted with anti-CCP and IgM RF is associated with more pronounced destructive changes in the joints.

## 1. Introduction

Rheumatoid arthritis (RA) is one of the most prevailing inflammatory arthropathies that affects primarily able-to-work persons what attributes a high social significance to the disease. Destruction of bone and cartilage is one of the key manifestations of RA [1]. The development of RA is associated with the formation of a wide spectrum of autoantibodies, including rheumatoid factors (RFs) and anti-citrullinated protein antibodies (ACPAs) (antiperinuclear factor, antikeratin antibodies, antibodies to cyclic citrullinated peptide (anti-CCP), modified citrullinated vimentin, anti-MCV, citrullinated fibrinogen, etc.), the presence of which contributes substantially to the course and prognosis of disease [2, 3]. At present, ACPA-positive and ACPA-negative RA subtypes are identified, which differ in molecular mechanisms of pathogenesis, severity course and approaches to the treatment administered [4–6]. ACPA-positive RA is characterized by accelerated radiologic progression, a severe course of the disease with higher total mortality [6, 7].

Hyperproduction of proinflammatory cytokines (interleukins (IL)-1, 6, 17, tumor necrosis factor *α*— TNF-*α*) and growth factors in RA promotes the liberation of proteolytic enzymes (matrix metalloproteinases—MMPs) by synovial cells causing the destruction of connective tissue of the joint [8] and stimulates an increased production of receptor activator of nuclear factor NF-*κ*B ligand (RANKL) [9, 10].

MMPs present a group composing over 20 proteolytic enzymes responsible for the cleavage of protein components of extracellular matrix. The family of MMPs includes collagenases (MMP-1, 8, and 13), which induce the degradation of collagen I, II, and III type, and stromelysins (MMP-3, 10, and 11), ensuring the proteolysis of noncollagen proteins (fibronectin, elastin) [11]. At present, MMP-3 is considered as one of key mediators of articular destruction in RA [12, 13].

RANKL [14] presents a soluble RANK ligand that occupies a central place in molecular regulation of bone tissue remodeling. RANKL is produced by synovial fibroblasts and activated T-lymphocytes. When binding with a RANK specific receptor located on osteoclasts precursors, RANKL stimulates osteoclastogenesis and bone resorption [14, 15].

An important factor in the formation of osteoclasts and acceleration of bone resorption in RA is presented by anti-MCV. The studies in vitro and in vivo have demonstrated the ability of anti-MCV to specifically interact with CV (citrullinated vimentin) expressing upon the membrane of osteoclast precursors [16–18]. The process induces the production of TNF-*α* and mediated by this cytokine expression of macrophage colony stimulating factor (MCSF) and RANKL, thus increasing osteoclast precursor sensitivity for differentiation into mature osteoclasts.

One of the markers of cartilage tissue destruction is the cartilage oligomeric matrix protein (COMP). COMP is a noncollagen glycoprotein of the thrombospondin family that is present in extracellular matrix of articular cartilage, synovium, and tendons [19]. The serum level of COMP [20, 21] correlates with radiologic changes in the joints, the concentration of C-reactive protein (CRP) and MMP-3 in blood and is a prognostic factor of severe destructive lesion to the joints in RA [21].

The objective of the study was to evaluate the relationships between the level of autoantibodies (IgM RF, anti-CCP, and anti-MCV) in blood serum, radiologic changes, and laboratory markers of bone and cartilage destruction in RA patients.

## 2. Materials and Methods

### 2.1. Patient Selection

A total of 114 patients, with definite diagnosis of RA, who were followed up at Nasonova Research Institute of Rheumatology, Moscow, within the period from 2009 till 2012, were examined. Candidate patients fulfilled the following criteria: (1) they were available for first posteroanterior radiographs of hands and anteroposterior radiographs of feet, (2) they had been treated with nonbiological DMARDs for more than 3 months before entry, and (3) they were not treated with biological DMARDs throughout the study. The control group included healthy donors matched for sex, age at the time of blood sampling, and area of residence.

The study was performed in accordance with the Declaration of Helsinki. All participating sites received approval from their governing institutional review board (or equivalent) and all patients provided written informed consent.

### 2.2. Disease Activity

We determined the DAS28, the Simplified Disease Activity Index (SDAI) score, and the Clinical Disease Activity Index (CDAI) score, using the following equations: DAS28 = 0.56 × $\sqrt{\left(\text{TJC}28\right)}$ + 0.28 × $\sqrt{\left(\text{SJC}28\right)}$ + 0.70 × ln⁡(ESR) + 0.014 × GH; SDAI score = SJC28 + TJC28 + PGA + EGA + CRP; and CDAI score = SJC28 + TJC28 + PGA + EGA, whereby the tender joint count (TJC) and the swollen joint count (SJC) were evaluated using 28 joints, global health (GH) was customarily replaced by the patient's global assessment (PGA) of disease activity (in millimeters on a visual analog scale [VAS]), scores for the PGA and the evaluator's global assessment (EGA) were measured in centimeters on a 0–100 mm VAS, the erythrocyte sedimentation rate (ESR) was measured as mm/hour, and the CRP level was measured as mg/dL. For all 3 composite measures, cut points for the disease activity states of remission (REM), low disease activity (LDA), moderate disease activity (MDA), and high disease activity (HDA) were determined. These established cut points are as follows: for the DAS28, REM < 2.6 ≤ LDA ≤ 3.2 < MDA < 5.1 < HDA; for the SDAI, REM ≤ 3.3 < LDA ≤ 11 < MDA ≤ 26 < HDA; and for the CDAI, REM ≤ 2.8 < LDA ≤ 10 < MDA ≤ 22 < HDA.

### 2.3. Biomarkers

ESR was determined by the Westergren method (normal value ≤ 30 mm/h). The serum concentration of CRP and IgM RF were measured by the immunonephelometric method. The normal level of CRP in blood serum was ≤5.0 mg/L. According to the manufacturer's instructions, the upper limit of normal (ULN) for IgM RF was 15.0 IU/mL. High-positive (>3 ULN; >45.0 IU/mL), low-positive (1–3 ULN; 15.0–45.0 IU/mL), and negative (≤ULN; ≤15.0 IU/mL) levels of IgM RF were identified. The quantitative estimation of anti-CCP was made by an electrochemiluminescent technique (ULN 17.0 U/mL) and enzyme-linked immunosorbent assay (ELISA) (ULN 5.0 U/mL). High-positive (>3 ULN; >50.0 U/mL—by an electrochemiluminescent technique, and >15 U/mL—by ELISA), low-positive (1–3 ULN; 17.0–50.0 U/mL and 5.0–15.0 U/mL, resp.), and negative (≤ULN; ≤17.0 U/mL and ≤5 U/mL, resp.) levels of anti-CCP were identified. The concentration of anti-MCV in blood serum was measured using ELISA kit. According to the manufacturer's instructions, the ULN for anti-MCV was 20.0 U/mL. High-positive (>3 ULN; >60.0 U/mL), low-positive (1–3 ULN; 20.0–60.0 U/mL), and negative (≤ULN; ≤20.0 U/mL) levels of anti-MCV were identified. COMP concentration in blood serum was measured in 34 RA patients by ELISA (ULN < 1185 ng/mL, in the serum of 30 healthy donors). The concentration of MMP-3 in blood serum was measured in 52 RA patients by ELISA with the use of commercial kits of reagents provided by “INVITROGEN” (ULN < 19.4 ng/mL, in the serum of 30 healthy donors) and “Bender MedSystems” in 28 RA patients (ULN < 9.3 ng/mL, in the serum of 24 healthy donors).

### 2.4. Radiographic Evaluation

Joint space narrowing and erosions were scored by the Sharp-van der Heijde (SvH) method.

### 2.5. Statistical Analysis

The results were statistically processed with the use of software package “Statistica 8.0” (“StatSoft,” USA), including commonly used methods of parametric and nonparametric analysis. For the parameters with distribution differing from normal, when two groups were compared, the Mann-Whitney *U* test was applied, whose results were presented as median (Me) with an interquartile range (IQR, from 25th to 75th percentile). The correlation analysis was performed according to the Spearman method. The differences were statistically significant at *P* < 0.05.

## 3. Results 

Baseline characteristics of 114 patients are given in Table 1.

As demonstrated by Table 1, the majority of patients were women of middle age with a prolonged course of disease, positive for IgM RF (82.5%), anti-CCP (83.3%), and anti-MCV (86.9%), and they had a high clinical (DAS 28 > 5.1) and laboratory (ESR > 35 mm/h, CRP > 20 mg/L) disease activity, X-ray phase II (43.9%). The patients received disease-modifying antirheumatic drugs; the main of which were methotrexate and glucocorticoides (GC) (61.4%).

Among RA patients, DAS 28: 5.9 (5.2–6.7), SDAI: 34.4 (23.5–45.5), and CDAI: 30.5 (20.9–41) corresponded to a high disease activity. An increased level of CRP and ESR values was recorded in 100 (87.7%) and 70 (61.4%) patients, respectively. A positive correlation of the level of IgM RF with DAS 28 (*r* = 0.3; *P* = 0.02), SDAI (*r* = 0.3; *P* = 0.02), and ESR (*r* = 0.3; *P* = 0.003) and the concentration of anti-MCV with SDAI (*r* = 0.25,   *P* = 0.02) and CDAI (*r* = 0.3,   *P* = 0.02) were identified. No significant correlation relationships of the anti-CCP level with indicators of disease activity have been identified.

### 3.1. Score of Radiological Data

The median of total modified Sharp score (mTSS), erosion score, and joint space narrowing score (JSN) amounted to 89 (57–117), 9 (3–27), and 76 (51–99), respectively. Radiographic changes were analyzed in groups of patients depending on the level of IgM RF, anti-CCP, and anti-MCV. No differences in radiologic signs of articular destruction in groups of RA patients high-positive and negative/ low positive for IgM RF and anti-CCP have been identified (*P* > 0,05) (data not shown). Among patients with high-positive level for anti-MCV, the higher values of mTSS and JSN were observed as compared with these indicators in anti-MCV negative/low positive RA patients (*P* < 0.05) (Table 2).

### 3.2. Laboratory Parameters

The level of COMP in RA patients did not differ from that in healthy donors: 472.5 (375.0–600.0) ng/mL *n* = 34 and 550.0 (435.0–740.0) ng/mL, respectively,* P* > 0.05. Alongside with that, a positive correlation between COMP concentration and anti-CCP levels (*r* = 0.4,   *P* = 0.02), anti-MCV (*r* = 0.5,   *P* = 0.002), X-ray phase (*r* = 0.4, *P* = 0.02) have been identified. In the group of patients with radiologic stages I and II, the level of COMP was lower than that in patients with RA stages III and IV,* P* < 0.05 (435.0; 367.5–472.5 and 550.0; 375.0–780.0, resp.).

The level of MMP-3 among RA patients was higher (35.0; 12.5–66.5 ng/mL, *n* = 52) in comparison with the group of healthy donors (7.7; 5.5–11.8 ng/mL,* P* < 0.05). A positive correlation of the level of MMP-3 with ESR (*r* = 0.4,* P* = 0.01) and CPR concentration (*r* = 0.4,* P* = 0.001) in blood was observed.

### 3.3. Relationship between the Level of Autoantibodies, Disease Activity, and Laboratory Parameters

**Figure 1:**
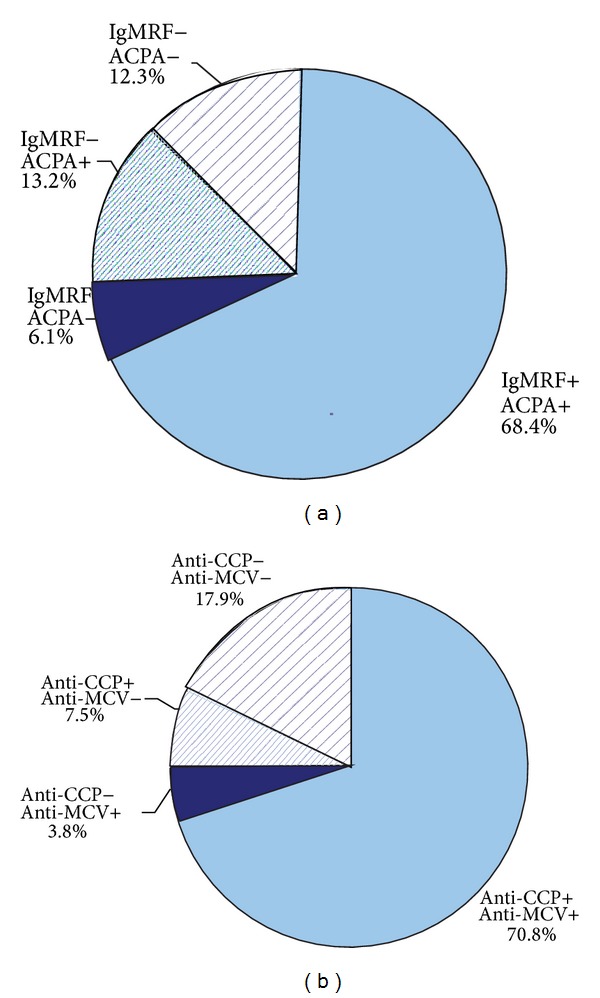
Dividing patients into groups, depending on the level of antibodies; “+”: high-positive level and “−”: negative/low positive level.

**Figure 2:**
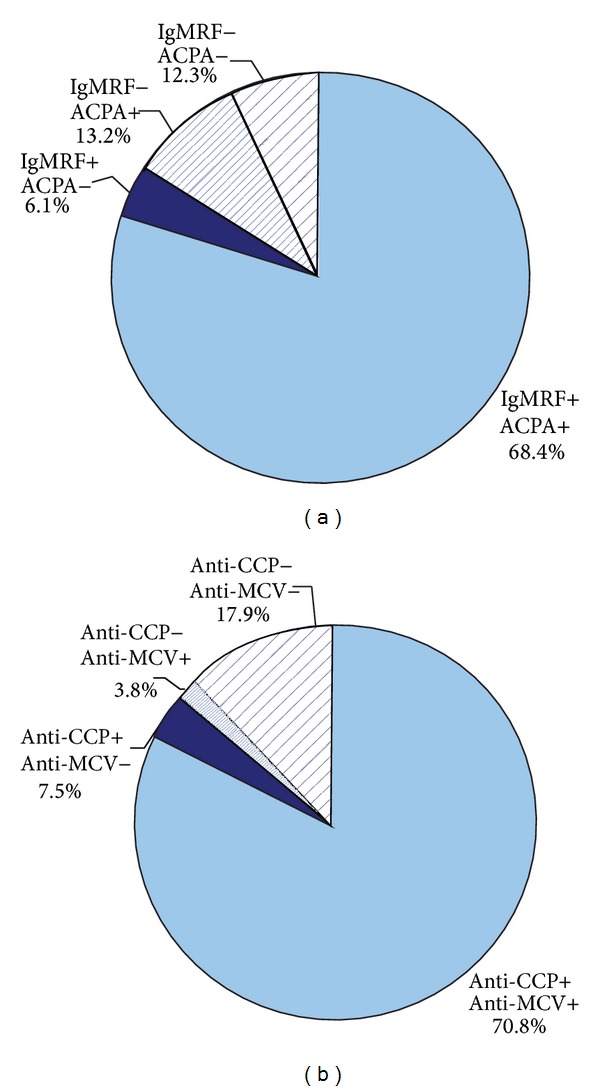
Dividing patients into groups, depending on the level of antibodies; “+”: positive level and “−”: negative level.

Depending on the positivity level for IgM RF, anti-CCP, and anti-MCV, all patients were divided into groups: high-positive (*n* = 84) and negative/low positive (*n* = 30) for IgM RF; positive (*n* = 94) and negative (*n* = 20) for IgM RF; high-positive (*n* = 89) and negative/low positive (*n* = 25) for anti-CCP; and positive (*n* = 95) and negative (*n* = 19) for anti-CCP. Groups of patients, divided according to the level of autoantibodies, are shown in Figures 1 and 2.

No differences in demographic indicators, administration of DMARDs and glucocorticoides, disease activity, and the level of laboratory biomarkers in groups of RA patients positive for IgM RF and anti-CCP have been identified (*P* > 0.05) (data not shown). Among high-positive anti-MCV patients (*n* = 79), the higher values of mTSS and JSN and the increased levels of COMP, ESR, IgM RF and anti-CCP were observed as compared with these indicators in anti-MCV negative/low-positive (*n* = 27) RA patients (*P* < 0.05) (Table 2). Besides, an increased concentration of MMP-3 was recorded more frequently in the group of high-positive anti-MCV patients (*n* = 64) as compared with that in patients with negative/low-positive anti-MCV titres (*n* = 16) (in 56.0% and 31.0% of cases, respectively,* P* = 0.038).

## 4. Discussion

Patients who are ACPA positive are especially prone to rapid radiographic progression, [22–26] and early aggressive treatment will particularly slow progression in those patients [27]. Less is known about the importance of the subtypes of ACPA related to prognosis. Our results also confirm that there are relationships between hyperproduction of ACPA (primarily anti-MCV), radiologic signs of erosive lesion to joints, and an increased level of markers of bone and cartilage destruction in RA. Our study shows that the presence of antibodies towards citrullinated vimentin is a stronger predictor of radiographic progression, compared with anti-CCP and RF. Patients who are anti-MCV positive are especially prone to rapid radiographic progression and need early aggressive treatment. Similar evidence on higher contribution of anti-MCV into development of destructive changes in the joints was obtained by Syversen et al. [28], while evaluating the prognostic value of anti-CCP and anti-MCV in 238 RA patients. The 10-year follow-up of anti-MCV-positive patients has demonstrated a higher incidence rate of radiologic progression (by total modified Sharp score) as compared with anti-CCP-positive patients (7.3 and 5.7 respectively,* P* < 0.01). Radiologic progression depended on the level of anti-MCV and reached maximum values in patients with concentration of this indicator higher than 254 U/mL. On a large group of RA patients (*n* = 273), Mathsson et al. [29] have also demonstrated a high rate of progression of destructive changes in the joints (by the change in Larsen score) in anti-MCV-positive patients. According to our data, high-positive levels of anti-MCV are associated with higher concentrations of markers of bone and cartilage destruction in blood serum (COMP, MMP-3), as well as with more pronounced destructive changes in the joints confirmed by radiography. When analyzing the groups of patients who were high-positive and negative/low-positive for anti-CCP, no significant differences have been obtained in COMP concentration and MMP-3 (*P* > 0.05). Among high-positive anti-MCV patients, higher levels of IgM RF and anti-CCP were recorded significantly more frequently, what may provide an additional impact on the rate and intensity of destruction of the joints in this group of patients. The association between ACPA status and radiographic progression is well established, [22–26] but the mechanisms behind this association are not known. ACPAs have been shown to contribute to disease progression by amplifying inflammation and damage in an animal disease model [30]. Alternative explanations for a more aggressive disease course in patients with high levels are that high ACPA levels could reflect either a more evolved immunological response or, as reported from a genome wide association study, a different genetic background [31]. According to our data, anti-MCV affects bone and cartilage destruction in a higher degree as compared with anti-CCP. Probably, the reason of such differences lies in different origin of ACPA. Vimentin presents a natural citrullinated protein that is synthesized and modified in macrophages of a synovial sheath affected by proinflammatory cytokines. CCP_2_ unlike the vimentin has synthetic origin. The evidence of pathogenetic relationships between anti-MCV and activity of osteoclasts in RA patients has also been obtained. Harre et al. [16, 17] have demonstrated a decreased level of PAD4 with a parallel increase in expression of PAD2 and CV in the process of differentiation of osteoclast precursors into mature cells. The laser scanning microscopy technique has revealed a direct binding of serum anti-MCV with CV on the surface of osteoclast precursors inducing a dose-related stimulation of bone resorption and osteoclastogenesis. An adaptive transfer of human affine-purified anti-MCV induced in mice Rag 1^−/−^ an increased number of osteoclasts in metaphyses of bones, an increased serum concentration of TNF-*α* and CTX1 with the unchanged level of osteocalcin in blood, and an increased number of CD11b^+^CD14^+^ osteoclast precursors of the spleen with hyperexpression of membrane receptors for MCSF and RANKL.

The level of COMP in our RA patients did not differ from that in healthy donors. This was probably associated with long disease duration in our patients. Recently, serum COMP was evaluated in patients with RA and was found to be preferentially elevated not in late-stage RA but in early-stage RA [24]. We have found a positive correlation between COMP concentration, anti-CCP levels (*r* = 0.4, *P* = 0.02), anti-MCV (*r* = 0.5,   *P* = 0.002), and radiologic stage of disease (*r* = 0.4,   *P* = 0.02) and higher levels of COMP in patients with III and IV X-ray phases. Similar evidence was obtained by Fujikawa et.al. In patients with early RA (*n* = 98), COMP values were statistically high in subjects positive for bone erosions on MRI (12.0 U/L) compared with the subjects who were negative for bone erosions (10.16 U/L,* P* = 0.017) [32]. Also several authors have found a positive correlation between COMP level and delta Larsen score [20, 21].

Besides, a number of authors have demonstrated correlations between the level of anti-MCV and disease activity [33, 34]. Also hyperproduction of anti-MCV is associated with higher clinic-laboratory activity of disease (DAS 28, ESR, CRP) and an increased rate of exacerbations per year [35]. Anti-CCP is less dependent on the clinical and laboratory disease activity [5, 36]. Our study has revealed a positive correlation of the anti-MCV level with indicators of disease activity, SDAI and CDAI, and an increase in ESR values in patients who are high-positive for anti-MCV. Level of anti-CCP was not correlated with disease activity.

The present study has some limitations. This is not a longitudinal study; data and blood samples were only collected and analyzed at one time point. It may be assumed that the detection of anti-MCV could be a consequence of destructive events, rather than a cause. However, given the data of other authors (that have looked at anti-MCV as a predictive marker of erosions), this alternative hypothesis could likely be discarded.

## 5. Conclusions

Thus, the results of our study have suggested that there is relationship between the level of autoantibodies in blood serum and the development of destructive changes in the joints. Anti-MCV affects bone and cartilage destruction in a higher degree as compared with anti-CCP and IgM RF. Patients who are anti-MCV positive are especially prone to rapid radiographic progression and need early aggressive treatment.

## Figures and Tables

**Table 1:** Demographic, clinical, and laboratory data for the group of RA patients (*n* = 114).

Parameters	Value
Number of females/males	93/21
Age, years Me (IQR)	53.0 (42.0–60.0)
Disease duration, months, Me (IQR)	60.0 (28.0–108.0)
X-ray phase, *n* (%)	
I/II/III/IV	2 (1.8)/50 (43.9)/46 (40.4)/16 (14)
DAS 28, Me (IQR)	5.9 (5.2–6.7)
DMARD use, *n* (%)	109 (95.6)
MTX use, *n* (%)	86 (75.4)
ESR, mm/hr Me (IQR)	38.0 (29.0–60.0)
CRP, mg/L Me (IQR)	22.4 (12.7–47.2)
IgM RF positivity, *n* (%)	94 (82.5)
Anti-CCP positivity, *n* (%)	95 (83.3)
Anti-MCV positivity (*n* = 106), *n* (%)	92 (86.9)

**Table 2:** Characteristics of RA patients depending on the level of positivity for anti-MCV, Me (IQR).

Parameters	High-positive anti-MCV (>60.0 U/mL) (*n* = 79)	Negative/low-positive anti-MCV (≤60 U/mL) (*n* = 27)	*P*
Age, years	53.5 (44.5–62.5)	48.0 (35.5–59.0)	>0.05
Disease duration, months	60.0 (28.5–111.0)	72.0 (33.0–121.0)	>0.05
Number of females/males	62/17	24/3	>0.05
DMARD use (MTX/other DMARDs), %	79.7/13.9	81.5/18.5	>0.05
DAS 28	5.9 (5.1–6.7)	5.8 (5.1–6.4)	>0.05
ESR, mm/hr	40.0 (30.0–61.0)	30.0 (21.0–48.5)	0.02
CRP, mg/L	23.1 (12.4–48.2)	18.4 (10.5–37.0)	>0.05
COMP, ng/mL	485.0 (405.0–655.0) *n* = 27	340.0 (270.0–535.0) *n* = 7	0.02
Elevated levels of MMP-3, %	56.0 *n* = 64	31.0 *n* = 16	0.038
Negative/low-positive IgM RF, *n* (%)	14 (17.7)	15 (55.6)	<0.01
High-positive IgM RF, *n* (%)	65 (82.3)	12 (44.4)	<0.01
Negative/low-positive anti-CCP, *n* (%)	4 (5)	19 (70.4)	<0.01
High-positive anti-CCP, *n* (%)	75 (95)	8 (29.6)	<0.01
Joint space narrowing (JSN)	82.0 (60.0–105.0)	50.0 (29.0–82.0)	<0.01
Total modified Sharp score (mTSS)	96.5 (65.0–122.0)	57.0 (31.0–88.0)	<0.01
